# A configurational analysis on career success of scientific and technological innovation talents in universities

**DOI:** 10.3389/fpsyg.2022.1068267

**Published:** 2022-12-08

**Authors:** Guoli Qu, Bingyue Sun, Dan He, Xingxing Guo

**Affiliations:** ^1^School of Economics and Management, Northeast Electric Power University, Jilin, China; ^2^School of International Business, Jilin International Studies University, Changchun, China

**Keywords:** social capital, human capital, psychological capital, universities, career success, fuzzy set qualitative comparative analysis (fsQCA)

## Abstract

**Introduction:**

Increasing the career success of scientific and technological innovation talents has become an important means of keeping and using talents in countries around the world. However, the problem has not been solved effectively.

**Methods:**

Thirty-five cases were chosen in this study. The combined effects of human capital, psychological capital, micro-social capital, team social capital, and macro-social capital on the career success of scientific and technological innovation talents, as well as relevant influencing mechanisms, were discussed using qualitative comparative analysis (QCA).

**Results:**

Results demonstrate that: (a) while a single factor cannot be the only condition for the high career success of scientific and technological innovation talents in universities, increasing macro-social capital plays a relatively universal role in a high career success rate. (b) There are three paths driving the high career success of scientific and technological innovation talents in universities. (c) There are two paths driving the non-high career success of scientific and technological innovation. Both have characteristics of absent psychological capital and micro-social capital. Moreover, the driving mechanism of high career success has an asymmetrical causal relationship.

**Discussion:**

Research conclusions are not only conducive to expanding the research perspectives of social capital theory and career success, but also provide valuable insight into how to stimulate the career success of scientific and technological innovation talents in universities.

## Introduction

Scientific and technological innovation is an important indicator of scientific and technological strength, and even the comprehensive strength of a country or a region. It is the core competitiveness of national development and plays a critical role in improving the scientific and technological innovation ability of a country. As the main carriers of scientific and technological innovation, scientific and technological innovation talents in universities are the valuable wealth of a country or a region. Training and retaining scientific and technological innovation talents have become the primary task for countries to implement innovation-driven strategies and promote high-quality development. Talent development is closely related to career planning. It is necessary to promote the career success of scientific and technological innovation talents in order to train and retain them. Universities are the cradle of scientific and technological innovation talents and an important base for the transformation of scientific and technological achievements. The career success of university scientific and technological innovation talents, especially the objective career success, is directly reflected in the output of scientific and technological achievements. Therefore, it is of direct and important significance to study their career success stimulation paths in universities to promote scientific and technological progress.

## Literature review and model construction

Studies on career success can be divided into two parts: one is studies on the conception and measurements of career success, the other part is on its the influencing factors.

### Definition of career success

Studies focusing on career success started in the 1980s. At that time, scholars believed career success to be the “positive psychological feeling or related work achievement that one person has accumulated gradually (London and Stumpf, 1982)”. Such a definition has been extensively applied (Seibert et al., 1999). With continuous deepening studies, relevant studies further divided career success into objective and subjective success (Gattiker and Larwood, 1988; Judge and Bretz, 1994; Kuijpers et al., 2006). Objective success is the indicator that can be observed externally and quantified, such as material success (e.g., salary and property), social class (e.g., status and title), influential force, etc. (Dries et al., 2009). Subjective success refers to the career satisfaction achieved by individuals inside or outside and is a subjective evaluation of individuals (Greenhaus et al., 1990). Subjective success and objective success are two aspects of career success. They interact mutually, and none is dispensable (Zhao, 2007). For individuals, evaluation standards of objective and subjective career success are not always overlapped. The objective career success is not the reason for the subjective one. A high salary and high status may not mean high satisfaction and high sense of achievement (Korman et al., 1981). Hence, scholars suggest simultaneously investigating objective and subjective aspects of career success in studies.

The late 1990s ushered in the era of a knowledge-driven economy, and changes in external environments, especially organizational environments, profoundly influenced the career life of employees. The contractual relationships between organizations and employees are not single and fixed. The mobility and unpredictability between employees and organizations increased, and the era of “Boundaryless Career” arrived (Arthur and Rousseau, 1996). Under this circumstance, connotations of career success changed, and it became very difficult to find a unified objective standard to measure success or the lack of it (Yan et al., 2008). On this basis, Eby et al. (2003) suggested using the existing subjective indicator (career satisfaction) and objective indicators (competitiveness of employees in labor markets in and out of organizations) as a new comprehensive indicator of career success. Such opinion replaced the original objective indicators (e.g., income and social status) with the competitiveness indicator, which better conformed to the connotations of boundaryless career. Hence, it has been approved by more scholars (Zhao, 2007; Yan et al., 2008).

Additionally, Eby et al. (2003) designed an occupational competitiveness questionnaire, which contained six problems of two dimensions: intra-organization competitiveness and inter-organization competitiveness. The career satisfaction questionnaire compiled by Greenhaus et al. (1990) and consisting of five items, is mostly used for the career satisfaction scale. Long (2002) revised the career satisfaction questionnaire according to Chinese situations. On this basis, Wang and Long (2009) revised and tested the career success questionnaire combined with the career competitiveness questionnaire and career satisfaction questionnaire, and showed that the questionnaire has good structure validity, reliability and calibration validity, which can be used as a measurement tool for relevant empirical research.

### Influencing factors of career success

There are many studies on the influencing factors of career success. Many empirical research results and meta-analysis studies have proved that relevant demographic variables (Judge et al., 1995), human capital characteristic variables (Boudreau et al., 2001), personality trait variables (Ng et al., 2005), and organizational variables (Abele and Spurk, 2009) play significant roles in predicting career success. Zhou et al. (2015) believed that the influencing factors of career success can be divided into human, psychological and social capital from the perspective of capital theory. Moreover, a comprehensive model of the influencing factors of individual career success was constructed from the standpoint of capital theory.

(1) Human capital influences career success. Turner (1960), an American sociologist, proposed two pathways to realize social mobility. The competition-mobility opinion believed that a career can be viewed as a completely open market, where everyone can move upward and achieve an elite status with their talents and efforts (Turner, 1960). Individuals must improve their skills through continuous self-efforts to compete with others in the market. Therefore, people with more knowledge and skills find it easier to achieve career success and attain the status of social elites (Rosenbaum, 1984). Since human capital can bring high rewards to individuals (Becker, 1964), it has positive influence on career success (Zhou et al., 2015).

Relevant scholars have been testing the influences of human capital on career success through empirical studies since the 1990s. Keeton (1996) analyzed the characteristics of successful female managers in government sectors and found that education background, intelligence, and working skills in human capital all led to their career success to different extents. Sandy et al. (1999) conducted a quantitative study on employees of different types of enterprises and found that working time, working experiences, education background, and job engagement in human capital were all highly related to their career success. This conclusion has been proved by different researchers. Through an empirical study, Gerard (2003) found that human capital elements like education level and quality, years of working, and working experiences all had positive correlations with employee career success. Dreher and Ash (1990), Tharenou (1997), and Kirchemeyer (1998) discovered from empirical studies that education, working experiences, and years of work are the most favorable factors in predicting the career success of individuals.

(2) Psychological capital influences career success. Psychological resources theory (Hobfoll, 2002) believes that high-level psychological capital can encourage employees to realize their goals and achieve career success. Luthans et al. (2007a) proposed the framework of psychological capital state (POB framework) for the first time. The POB framework deems that psychological capital can be divided into four states; hope, optimism, toughness, and confidence. Subsequently, scholars have carried out studies according to the POB framework, which focuses on the influences of psychological capital on organizational performances, employee attitude, and behaviors. Many studies have proved that psychological capital has a positive effect on organizational performance. For instance, positive psychological capital can promote task performance and the surrounding performance of employees (Zhong, 2007). Luthans et al. (2005, 2007a, 2008) proved that positive psychological capital had positive correlations with performance pay, superior evaluation, and job satisfaction.

Currently, many studies discuss the relationship between psychological capital and career success independently. The job performance of employees may influence objective career success directly, and job satisfaction is an important indicator of career success (Zhou et al., 2015). Larson and Luthans (2006) demonstrated that all four positive psychological states could significantly improve the job satisfaction of employees. They believed that such phenomenon could be interpreted as follows. If employees have positive psychological states, they generally believe that they can eventually achieve career success and thereby keep relatively high enthusiasm and momentum to work. They typically persist in work upon setbacks and difficulties. As a result, psychological capital can influence career success (Zhou et al., 2015).

(3) Social capital influences career success. Baker (2002), an American scholar, pointed out that success depends on the human and social capital of individuals. According to the sponsorship-mobility opinion of career success (Turner, 1960), people with support from the elite class in an organization find it easier to achieve career success, because they possess more organizational resources and career development opportunities. Scholars have conducted empirical studies on the influencing mechanism of social capital on career success. To be the most representative, Seibert et al. (2001) proved that social network structure could positively predict social resources, and social resources could promote career success through positive influences on the social network effect. This result was based on the questionnaire survey data of 448 students in a university. Meanwhile, they proposed a mediation model for social capital influencing career success.

Hananifan used the term, “Social capital” for the first time (Hanifan, 1916). He elaborated on the importance of social communication to education, and community society through social capital. However, the concept of social capital was not so explicit at that time. Bourdieu (1986), a French sociologist, was the first to define the concept of social capital and apply it to the sociology field. He believed that social capital is the practical or potential resource entity, and it has two characteristics. Firstly, social capital is a kind of resource connected to the qualification of group members and social networks. Secondly, social capital connects the relational networks of social members who know each other and have similar cognition. Therefore, Bourdieu’s concept focuses on the microscopic individual.

Coleman (1990) inherited and absorbed some of Bourdieu’s (1986) opinions when he studied social capital. However, Coleman (1990) expanded the concept of social concept to the meso-level and provided a more extensive understanding of social capital. Based on the theory of rational behavior, he believes that social capital is not only a means to increase individual interests, but also an important resource for solving collective action problems.

In Making Democracy Work: Civic Traditions in Modern Italy, Putnam et al. (1993) defined social capital as a characteristic, i.e., trust, network, norms, of social organizations. These features improve social efficiency by promoting the cooperative actions of people. In this manner, Putnam et al. (1993) concept of social capital has exceeded the research scopes of Bourdieu (1986) and Coleman (1990), which has increased to the macroscopic level.

To sum up, existing studies on the influencing factors of career success mainly focus on one or several simple factors. However, these studies only conducted dispersed studies of career success using single or several simple variables. The reliability of such research conclusions and their promotion and application values in management practices have decreased (Zhou et al., 2015). As per research methods, most used meta-analysis (Zhou et al., 2015) and empirical analysis (Wang and Long, 2009). As such, there are few QCAs of the collaborative interaction of factors on career success. This provides a space to improve the career success of scientific and technological innovation talents from the perspective of empirical analysis.

The QCA was introduced innovatively in this study. Influencing factors of the career success of scientific and technological innovation talents in universities were summarized from the perspective of capital theory. The combinations of influencing factors that generate high career success rate as well as the interaction relations of factors in the combination were recognized using tracing logics. This provides a granularity analysis of the relations of career success with human capital, psychological capital, and social capital.

**FIGURE 1:**
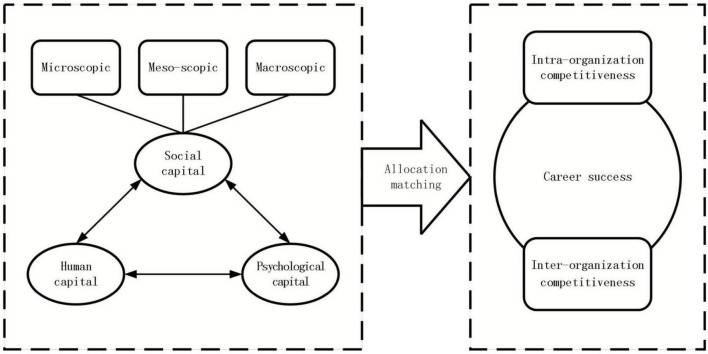
Theoretical model.

In conclusion, this study focuses on two causal relationships: (1) whether human capital, psychological capital, and social capital are essential conditions for scientific and technological innovation talents to achieve a high career success rate. (2) How to couple these capital elements to achieve high career success rate of scientific and technological innovation talents. The theoretical model in this study is shown in Figure 1.

## Materials and methods

### Qualitative comparative analysis

Traditional quantitative analyses typically believe that independent variables are mutually independent and establish regression equations on this basis for the linear test of causal relationships. In fact, variables in the career success of scientific and technological innovation talents influence mutually rather than being independent. They affect the career success of scientific and technological innovation talents in universities together. On this basis, qualitative comparative analysis (QCA) was applied as the major research method in this study.

Qualitative comparative analysis was proposed by Ragin, a sociologist, in the 1980s. Unlike the traditional quantitative analysis, QCA emphasizes the analysis of a combined effect rather than a net effect. Through cross-case comparison, researchers can recognize the combination of conditions that may lead to the occurrence or non-occurrence of outcomes, thus enabling them to find logical relations between condition matching modes and outcomes. Therefore, QCA can further recognize the collaborative effect of multiple conditional variables under the admission of complexity of causality (Du and Jia, 2017). Compared to traditional quantitative studies and qualitative studies based on case analysis, QCA was chosen as the research method in this study for the following reasons. First, QCA breaks the limitation arising from the interdependence analysis of several conditions in the past. From the perspective of combination, QCA fully explores the influences of the collaborative effect of several conditions on the career success of scientific and technological innovation talents from three capital theories. This is conducive to disclosing the complicated collaborative influencing mechanism of multiple factors on the career success of scientific and technological innovation talents. Second, QCA has no limitation in sample size. It is not only applicable to studies with large sample sizes, but also to studies with medium and small sample sizes. Through the cross-case comparison of samples, researchers have recognized combinations of conditional variables and logic behind the outcome variable, providing a deeper qualitative analysis and guaranteeing external promotion to some extent. Third, QCA can recognize different equivalent condition combinations that lead to the outcome. This helps us understand the differential driving mechanism in different scenes and further discuss the matching and substitution relations among conditions. Moreover, fsQCA, based on Boolean algebra, does not cause variable missing-induced deviation. Therefore, it needs no control variables (Fainshmidt et al., 2020).

Qualitative comparative analysis includes fuzzy set qualitative comparative analysis (fsQCA), multi-value set qualitative comparative analysis (mvQCA), and clear set qualitative comparative analysis (csQCA). Among them, mvQCA and csQCA only apply to category problems with discontinuous data. However, fsQCA does not only process category problems, but is also used to process some membership problems in this study. As a result, fsQCA was chosen as the research method in the present study.

### Data

Based on the case selection requirements of the QCA research method, the homogeneity of the background information of the cases is used to reduce the influence of the control variables, and the variability of explained variables between cases results in different configurations. Besides, Central Committee and The State Council on Further Strengthening Talent Work identified the talents as people with some knowledge or skills to make creative labor activities; promote the development of socialism, material civilization, political civilization, and spiritual civilization; and make positive contributions to building socialism with Chinese characteristics. According to this regulation, talents with good scientific and technological innovation ability, who participate in scientific and technological innovation activities directly, and make important contributions to technological development and social progress, are the scientific and technological innovation talents (Wang and Lin, 2008). With reference to this definition and standards proposed by Tan et al. (2004), 35 talent cases were chosen in Northeast Electric Power University in China. The data was collected by sending questionnaires directly to the subjects from April 20, 2021 to May 30, 2021. The dateset is shown in Supplementary Data Sheet 1. The descriptive statistics of the samples are shown in Table 1.

**TABLE 1:** Descriptive statistics of the sample.

	Sample (*N* = 35)	Percentage (%)
**Sex**		
Male	19	54%
Female	16	46%
**Age**		
30–35	2	6%
35–40	17	49%
40–45	11	31%
45–50	5	14%
**Seniority**		
0–5	2	6%
5–10	14	40%
10–15	17	48%
15–20	2	6%
**Research area**		
Electrical engineering	8	23%
Chemical engineering	13	37%
Construction engineering	11	31%
Math	3	9%
**Title**		
Professor	22	63%
Associate professor	13	37%
**Marital status**		
Married	35	100%
Unmarried	0	0
**Highest education**		
Doctor degree	35	100%
Master degree	0	0
Other	0	0

## Measurements and calibration of variables

The principles of scale selection in this study are as follows: first, the scale content is highly compatible with the scenario of this study; second, the scales have high reliability and validity in the reviewed literature.

### Outcome variable

The measurement of career success adopted the scale developed by Wang and Long (2009), which includes two sub-scales: career satisfaction and career competitiveness. Furthermore, the sub-scale of career competitiveness also includes two dimensions: the internal competitiveness of the organization and the external competitiveness of the organization. Therefore, the career success questionnaire consists of three dimensions, namely, the internal competitiveness of the organization, the external competitiveness of the organization and the career satisfaction. A total of 11 questions were included, and the career success of the sample in this study was measured by the sum of scores of the 11 questions in the questionnaire.

The Cronbach’s coefficients of the two sub-scales were 0.739 and 0.753, respectively, indicating high reliability. In addition, this questionnaire includes not only objective indicators (career competitiveness), but also subjective indicators (career satisfaction), which is more in line with the career success concept in the theory of the “Boundaryless Career.” Therefore, this study adopted this scale to measure the variable of career success.

### Antecedent variables

#### Human capital

At present, the most commonly used human capital measurement questionnaire in academia is the human capital characteristic scale designed by Lepak and Snell (2002), which includes two subscales: human capital value subscale and human capital uniqueness subscale, and the Cronbach’s coefficients were 0.90 and 0.85, respectively. There are 22 questions in total, using Likert’s five points to score. The original questionnaire was in English, and was translated into Chinese in this study. The final human capital score of the sample was scored by the sum of the scores of the 22 questions. Human capital in this questionnaire mainly refers to the labor ability of employees, including the irreplaceable ability of the individuals with the labor value, which is consistent with the concept of the human capital in this paper. Therefore, this questionnaire was adopted to measure the human capital in the research.

#### Psychological capital

Psychological capital is a positive psychological state manifested by individuals in the process of growth and career development, and mainly includes four aspects: self-confidence, optimism, resilience and self-efficacy (Luthans et al., 2006). The psychological capital of this questionnaire is embodied in: having confidence and making the necessary efforts to achieve success in the face of challenging work (self-efficacy); having a positive attribution of present and future success (optimism); perseverance toward goals, and being able to adjust the path to achieve them when necessary to achieve success (hope); persevering, recovering quickly, and growing to succeed when faced with adversity and problems (resilience). This is consistent with the general psychological capital questionnaire of Luthans et al. (2007a). There are 24 questions in total, and the score is scored by the Likert six-point method. The psychological capital of the sample in this study was measured by the total score of the 24 questions in the questionnaire.

#### Social capital

Referencing Brown’s studies, social capital was divided into three dimensions, including microscopic dimension, mesoscopic dimension, and macroscopic dimension. These three dimensions correspond to individual social capital, team (organization) social capital, and macrosocial capital.

In order to be closer to the context of Chinese human relations society, this paper adopted the individual social capital scale developed by Wang (2006) to measure micro-social capital. The individual social capital scale includes six dimensions: network size, network differences, social resources, kinship support, friend support, and acquaintance support. The Cronbach’s coefficients of each dimension were 0.812, 0.810, 0.840, 0.843, 0.658, and 0.872, respectively, indicating high reliability. There are 23 questions in the questionnaire, which are scored with the Likert six-point method. The final score consists of the sum score of 22 questions.

Team social capital refers to a resource exchange capability embedded in the internal social network of team members. It mainly reflects the opportunity, willingness and ability of resource exchange. In order to better reflect the team social capital in the Chinese context, this paper adopted the team social capital scale of Ke et al. (2007). The scale includes six dimensions: colleague trust, network density, common language, interaction strength, common vision, and supervisor trust, and the reliability of each dimension is above 0.7. Network density and interaction intensity reflect the opportunity for resource exchange; colleague trust and supervisor trust reflect the willingness to exchange resources; common language and shared vision reflect the ability to exchange resources. The team social capital scale includes a total of 23 questions. Except for the three yes-no questions in the dimension of network density, the five-point Likert method was used to score. The final score consists of the sum of 23 questions.

Macro-social capital refers to the degree of individual perception of the norms, trust and other characteristics of the country. Measurement scales of macro-social capital are relatively rare. In order to be closer to the definition of macro-social capital in this paper, we adopted the measurement method in the World Trust Survey (WVS) and the CGSS survey to measure macro-social capital. That is: “Do you think most of the people around you can be trusted?” Likert six-point method was used to evaluate.

For the adopted foreign scales, in order to avoid the differences caused by semantic differences and cultural backgrounds, this study invited doctoral students in English major to perform translation-back translation. Before the formal investigation, five graduate students and five teachers were invited to conduct pre-investigation. The questions they brought up and possibly ambiguous sentences were adjusted again. The specific items finally determined are shown in Supplementary Appendix A.

### Validity and reliability analysis

Table 2 first shows that Cronbach’s coefficients of employee career success, human capital, psychological capital, microsocial capital and team social capital are higher than 0.8. This indicates that the questionnaire had good reliability. Secondly, validity of the questionnaire was tested by factor analysis. The Kaiser–Meyer–Olkin (KMO) test result was higher than 0.7, and the lowest cumulative variance contribution rate was 73.244%, indicating the good structural validity of the questionnaire. The composite reliability (CR) of factors was higher than 0.8 and the average variance of extraction (AVE) was greater than 0.6, indicating that the questionnaire had good convergent validity. AVE of factors was higher than the square of its correlation coefficients with other factors. This reflected that the questionnaire had good discrimination validity. In other words, the questionnaire had good reliability and validity and it could be used in further studies.

**TABLE 2:** Reliability and validity analysis.

Variables	Dimensions	Minimum factor load	Cronbach’s	CR	AVE	KMO value	Cumulative variance contribution rate (%)
Human capital	Value of human capital	0.544	0.929	0.945	0.754	0.726	76.792
	Uniqueness of human capital	0.519					
Psychological capital	Self-efficacy	0.526	0.876	0.964	0.535	0.663	80.640
	Hope	0.604					
	Toughness	0.580					
	Optimism	0.675					
Microsocial capital	Network size	0.821	0.931	0.980	0.670	0.713	83.645
	Network difference	0.516					
	Social resources	0.666					
	Support of relatives	0.773					
	Support of friends	0.755					
	Support of acquaintances	0.757					
Team social capital	Trust of colleagues	0.569	0.962	0.980	0.723	0.788	77.210
	Common language	0.827					
	Interaction strength	0.582					
	Shared vision	0.895					
	Trust of director	0.725					
Career success	Intra-organizational competitiveness	0.701	0.893	0.959	0.683	0.814	73.244
	Inter-organizational competitiveness	0.655					
	Career satisfaction	0.632					

### Variable calibration

Variable calibration is a special process of fsQCA, helping to transform the original variables into continuous membership fractions within 0–1. With reference to previous studies (Fiss, 2011; Greckhamer, 2016), the totally affiliated and totally unaffiliated crossing points between five antecedent variables and one outcome variable (career success) were set as the maximum, mean, and minimum of the descriptive statistics of case samples, respectively. The calibration anchoring points of variables are shown in Table 3.

**TABLE 3:** Set and calibration statistics.

Configuration	Fuzzy set calibration		
	Complete affiliated point	Intersection point	Complete non-affiliated point
Career success	26	38.91	50
Human capital	60	81.63	105
Psychological capital	62	87.63	111
Microsocial capital	51	76.51	105
Team social capital	54	87.71	109
Macrosocial capital	1	3.54	5

## Analysis results

### Necessity analysis

A superset of outcomes is a requirement. If a requirement is included in the truth table for analysis, it might be simplified as a “logic remainder term.” Requirements have to be first analyzed before the combination analysis (Charles and Du, 2019). Table 4 shows that the consistency of human capital, psychological capital, microsocial capital, team social capital, and macrosocial capital was less than 0.9. Hence, none of these five antecedent conditions is required for the career success of scientific and technological innovation talents. In other words, these five conditions are not bottlenecks of the career success or non-career success of scientific and technological innovation talents.

**TABLE 4:** Necessity test for the single requirement of QCA.

Configuration	High career success consistency	Not high career success consistency
Human capital	0.776	0.642
∼ Human capital	0.632	0.803
Psychological	0.823	0.585
∼Psychological	0.541	0.813
Microsocial	0.783	0.590
∼ Microsocial	0.596	0.825
Team social	0.835	0.565
∼ Team social	0.588	0.807
Macrosocial	0.852	0.712
∼ Macrosocial	0.548	0.724

### Configuration analysis

The combinations of conditions that attribute the high career success of 35 samples were analyzed using fsQCA3.0 software. These different combinations reflect different capital environments to realize the same outcome (high career success degree). According to Fiss (2011) and Du and Jia (2017), this study set the consistency threshold, case threshold, and PRI consistency (proportional reduction in inconsistency) as 0.8, 1, and 0.7, respectively. The truth table is shown in Table 5.

**TABLE 5:** Truth table.

Human capital	Psychology capital	Microsocial capital	Team social capital	Macrosocial capital	Career success
1	0	1	1	1	1
1	1	1	1	1	1
0	1	1	1	1	1
1	1	1	0	1	1
1	1	0	1	1	1
1	1	0	1	0	0
0	1	0	1	1	0
1	0	0	1	1	0
0	0	1	0	0	0
1	1	1	0	0	0
0	0	1	1	1	0
0	0	1	0	1	0
0	0	0	1	0	0
1	0	0	0	1	0
1	0	0	0	0	0
0	0	0	1	1	0
0	0	0	0	1	0
0	0	0	0	0	0

The combinations of outcomes were determined according to the intermediate solution. The center conditions and contributing conditions of each combination were gained by embedding the relations of the parsimonious and intermediate solutions (Dul, 2019). In QCA, center conditions are important conditions that cause the occurrence of outcomes, while contributing conditions are auxiliary conditions for outcomes (Benoit Lihaux et al., 2017).

Table 6 summarizes the four paths that can stimulate the high career success rate of scientific and technological innovation talents and the two paths that can simulate the non-high career success of scientific and technological innovation talents. The consistency of all six paths reached more than 0.8, showing a relatively high consistency (Ragin, 2008). Next, these six paths were analyzed in this study.

**TABLE 6:** Combinations of conditions for high and non-high career success rates.

Configuration	High career success solution				Not high career success solution	
	H1	H2	H3	H4	NH1	NH2
Human capital	●	●	●		⊗	
Psychological capital	●	●		●	⊗	⊗
Microsocial capital	•		●	●	⊗	⊗
Team social capital		•	●	•		⊗
Macrosocial capital	●	●	•	●		⊗
Consistency	0.953	0.929	0.955	0.950	0.890	0.926
Raw coverage	0.595	0.620	0.598	0.628	0.656	0.565
Unique coverage	0.025	0.051	0.029	0.059	0.124	0.033
Overall solution coverage	0.734				0.689	
Overall solution consistency	0.926				0.894	

●, existence of center conditions; ⊗, missing center conditions; ∙, existence of contributing conditions; •, missing contributing conditions.

#### Paths to generate high career success rate

H1, H2, H3, and H4 are sufficient conditions for scientific and technological innovation talents to achieve employee career success. In QCA, the overall coverage of solutions reached 0.734, indicating that these four paths explained the major reasons for the career success of most scientific and technological innovation talents (Vis and Dul, 2018). These four paths are analyzed in the following text:

(1) H1 is the individual-dominated path based on social trust. It indicates that scientific and technological innovation talents in universities have good individual qualities (e.g., high human capital and high psychological capital) and high trust in society (existence of macrosocial capital). If microsocial capital also exists, scientific and technological innovation talents can achieve high employee career success. For individuals, microsocial capital, i.e., the network relationship of individuals, is an important factor in achieving career success (Granovetter, 1973).

Baker (2002) an American scholar, proposed that individual career success depends not only on the individual’s ability, but also on their relations with others. He based this on theoretical studies and practice experiences over the years. Salary, promotion, and the performance of an individual are determined by their interpersonal relations and enterprise relation network to a very large extent. Long (2002) emphasized the importance of the microscopic interpersonal network of individuals to guarantee employee career success. He believed that the “relation” in self-career management does not occur by accident, but is a Chinese characteristic. Individual relation refers to microsocial capital. Furthermore, we can analyze microsocial capital from the perspective of China’s sociocultural characteristics and current social background: firstly, China’s traditional society is relation-oriented and ethics-centered, and this has been agreed upon in the academic circle (Fei, 1998). Although the modernization process of nearly 100 years has brought significant changes in China’s society, relations still take the dominant role in the social behaviors of people (Wang, 2006). Secondly, the existing social formal system is not perfect. Social capital is gained from social resource configuration and status, which plays an irreplaceable role. Social capital is not only a method to decide the social status of people, but also an important way for social resource allocation (Li, 1995).

In conclusion, the microsocial capital of individuals plays an important role in their career life. Some scientific and technological innovation talents have high quality, high irreplaceable ability, good psychological quality, and generally good trust in the society. Microsocial capital decides the career success of these individuals. Individual relations, especially the “weak relation,” decide whether they can achieve career success (Granovetter, 1973).

(2) H2 is the path driven by individual capitals and social trust under the assistance of platforms. Given the high team social capitals, scientific and technological innovation talents with good individual qualities (e.g., high human capital and high psychological capital) and high trust in the society (high macrosocial capital) can achieve high career success even when there is no high microsocial capital. Ke et al. (2007) defined team social capital as “a kind of resource exchange ability embedded into the internal social relation network of team members.” Through a series of empirical studies, Ke et al. (2007) demonstrated that good team social capital could positively affect team efficacy through knowledge sharing and knowledge integration. Good team efficacy positively influences the career success of individuals in the team. Similarly, the connotations of individuals-organizational fit theory (Cable and DeRue, 2002) mentions that a good fitting between individuals and the organization can be achieved when there is ability agreement between individuals and the organization as well as an agreement between organizational supply and individual demands. The high fitting between individuals and the organization positively influences job performances (Zhu and Chen, 2005) and behavior performance (Zhao, 2009) of employees in the organization. Job performance and behavior performance are important influencing factors of the career success of individuals. Therefore, high fitting between individuals and the organization can positively influence the career success of individuals.

If scientific and technological innovation talents join or form an excellent team, they can accomplish the team goal and improve the team performance through division of labor, cooperation, and knowledge-sharing in the team. In this case, individuals with high fitting with the organization can improve their job performances through high team performances, thus realizing the ultimate goal of employee career success.

In other words, a scientific and technological innovation team tends to accept talents with good human and psychological capital, high irreplaceable ability, and outstanding personal ability. These talents generally conform to the “individual-organization ability agreement and organizational supply-individual demand agreement.” The high fitting between individuals and organizations positively influences the job performances of scientific and technological innovation talents, thus enabling them to achieve employee career success. This also reflects the necessity for the coexistence of high human capital, high psychological capital, high team social capital and high macrosocial capital in H1. Additionally, high human capital, high psychological capital, and high macrosocial capital exist as center conditions, while team social capital exists as a contributing condition. This indicates that scientific and technological innovation talents who conform to this path pay prior attention to improving their personal abilities.

(3) H3 is the path driven by human capital and social capital under the assistance of social trust. (Human capital-driven path under the assistance of social support) H3 demonstrates that scientific and technological innovation talents can achieve career success if high human capitals and high social capitals (e.g., microsocial capital, team social capital and macrosocial capital) coexist. This mutually corroborates with the job demand-resource (JD-R) model (Demerouti et al., 2001). The JD-R model believes that any job characteristic can divide job requirements and working resources. Job requirements are defined as “job’s requirements on physiology and ability of individuals and the factors that can only be realized through individual efforts.” On the contrary, job resources are defined as “various positive factors of physiology, psychology, society, and organization in jobs which can promote the realization of career objective or promote individual growth and development” (Demerouti et al., 2001).

The “double-path hypothesis” of the JD-R model believes that continuous job requirements may consume individual resources and energies and cause losses, thus resulting in negative influences, such as a series of job burnout. The acquisition of job resources may stimulate the job motivation of employees and thereby induce the gain process, thus improving job efficacy (Zhang et al., 2017). Scientific and technological innovation talents have to enhance their human capitals to improve their competitiveness. As one element of job requirements, human capital will continue to consume individual resources and energies, thus causing the loss process. However, social capital (including microsocial capital, team social capital, and macrosocial capital) stimulates the job motivation of individuals and triggers the gain process as a type of positive job resource. Moreover, the “buffer hypothesis” in the JD-R model believes that job resources could buffer individual loss by job requirements. In other words, job resources can relieve the negative influences of job requirements. As a kind of job resource, social capital can relieve stress and the negative influences on the human capital improvement of scientific and technological innovation talents. Besides, the “response hypothesis” in the JD-R model points out that high job resources can be better transformed into high-level job performances under high job requirements. Under the high job requirements, employees can devote themselves to work more and deploy a lot of existing job resources to complete the job objective, thus getting more new resources. Scientific and technological innovation talents can only transform social capital better into high-level job performances under high human capitals. The human capital and social capital supplement each other, and neither is dispensable. Therefore, high human capital and high social capital must coexist in H4.

In the dual-path hypothesis in the JD-R model, the buffer hypothesis and response hypothesis verify the reasonability of H3. Given the high human capital, scientific and technological innovation talents can achieve career success if there is high social capital (microsocial capital, team social capital, and macrosocial capital) at the same time.

(4) H4 is the path where psychological capital and social capital act together. Under the coexistence of high psychological capital and high social capital (including microsocial capital, team social capital, and macrosocial capital), scientific and technological innovation talents can achieve employee career success, no matter what the human capital is. A positive psychological state positively influences social resources accumulated by individuals (Zhou et al., 2015). Turban and Dougherty (1994) deemed that employees with positive psychological states were more willing to initiate a “master-apprentice relationship,” thus gaining more vocational guidance. They find it easier to achieve employee career success. Moreover, developing social resources is an initiative behavior. Individuals with active personalities are more likely to build extensive social network services, such as seeking allies or building relations with people who have power and influential forces, to achieve career success (Thompson, 2005). Similarly, individuals with positive psychological states not only have social networks with larger scopes and higher supports, but also possess longer friendships and acquire more emotions, tools, and social resources (Zhou et al., 2015). Lyubomirsky and King (2005) analyzed and summarized many cases to determine the relationship between positive emotions and success. He found that individuals with positive psychological states can acquire more support from marriage, friends, colleagues and superiors in terms of emotions and work.

In conclusion, psychological capital positively influences individual social capital. As the intermediate variable, social capital also positively influences the career success of individuals. This proves that the coexistence of high psychological capital and high microsocial capital in H4 leads to career success.

Besides, most samples of H4 were aged between 30 and 40, and their years of working were less than 7 years. According to Greenhaus’s (2006) five-stage theory of career development, most samples of H4 were in the initial stage of career life.

H1 and H2 form the second-order equivalent combinations of four paths. The coverage of H4 reached 62.8%, ranking the top in the four paths. In other words, most scientific and technological innovation talents achieve career success through H4. This fully proves the important effects of psychological capital and social capital (including microsocial capital, team social capital, and macrosocial capital) on employee career success. In H4, the effect of human capital is not obvious. In the era of knowledge economy, traditional human capital has no advantages of competitiveness (Luthans et al., 2007b). In practical life, organizational or team requirements for human capital should not be too strict. It is possible to achieve career success as long as talents make full use of social relation networks and have support from excellent teams (Ke et al., 2007). It is important for young scientific entrepreneurs conforming to this path to make full use of surrounding microsocial relations and join teams with development potentials. Generally speaking, macrosocial capital occurs in four paths, even occurring as the center condition in H1 and H2. This indirectly proves the importance of macrosocial capital to the career success of scientific and technological innovation talents (Putnam et al., 1993). Macrosocial capital reflects not only the trust degree of a person to the society, but also the overall social institution and degree of standardization. It stipulates that the state and government should pay particular attention to the development of macrosocial capital, and view the improvement of social trust and promoting citizens to participate in networks and mutual norms as an important job objective that aims to promote the career success of individuals, especially scientific and technological innovation talents (Putnam et al., 1993). H1 and H2 show the mutual substitution between microsocial capital and team social capital. When individual advantages are relatively obvious, there is high human capital, psychological capital, and social trust. When microsocial capital is in the disadvantaged position, attention should be paid to power of the team. Individual goals can be realized by depending on excellent teams, enabling individuals to improve their job and relation performances. Such individuals can finally achieve employee career success. On the contrary, under the same prerequisites, individuals who are not in excellent teams have to explore individual microscopic social networks, especially weak relations. They have to acquire more social resources through surrounding industries, hierarchies and levels to achieve employee career success. It is important to note that if psychological capital and social capital cannot coexist, human capital still exists as the important center condition. This inspires scientific and technological talents to improve their human capitals continuously before the advantages of psychological capital and social capital become apparent.

#### Paths generating non-high career success

Paths generating non-high career success were tested in this study. There were two paths generating non-high career success (NH1 and NH2). NH1 shows that individuals cannot achieve career success even if they have high team social capital and macrosocial capital since there is no human, psychological, and microsocial capital. NH2 shows that under the absence of psychological capital and social capital, individuals cannot achieve career success no matter how high the human capital is. It was found that both NH1 and NH2 are characteristic of the coexistence of non-high psychological capital and non-high microsocial capital. In other words, individuals may find it impossible to achieve career success if they have insufficient psychological capital and the microsocial capital is not advantageous, no matter how high the human capital, team social capital, and macrosocial capital are.

### Robustness test

The robustness test is an important link of QCA (Dul et al., 2020). The present study tested the robustness of the combinations of conditions for the high career success of scientific and technological innovation talents. First, the consistency threshold was adjusted from 0.8 to 0.85. The combinations, which were consistent with the original combination, are listed in Table 7. Second, the PRI consistency increased from 0.7 to 0.75; the corresponding combinations are listed in Table 8. The original four combinations were changed to three, and the parameters of solutions were consistent with the original results. The quantity change of solutions is not used as the criteria of robustness. In different models (operations), the solution is considered robust if combinations and solution parameters are similar to the original model. Therefore, the robustness test shows that results are robust.

**TABLE 7:** The configuration after raising the consistency threshold.

Configurations	High career success			
	H1	H2	H3	H4
Human capital	●	●	●	
Psychological capital	●	●		●
Microsocial capital	•		●	●
Team social capital		•	●	•
Macrosocial capital	●	●	•	●
Consistency	0.953	0.929	0.955	0.950
Raw coverage	0.595	0.621	0.598	0.628
Unique coverage	0.025	0.051	0.029	0.059
Overall solution coverage	0.734			
Overall solution consistency	0.926			

●Existence of center conditions; •Existence of contributing conditions.

**TABLE 8:** The configuration after improving the PRI consistency.

Configurations	High career success		
	H1	H3	H4
Human capital	●	●	
Psychological capital	●		●
Microsocial capital	•	●	●
Team social capital		●	•
Macrosocial capital	●	•	●
Consistency	0.953	0.955	0.950
Raw coverage	0.595	0.598	0.628
Unique coverage	0.025	0.029	0.059
Overall solution coverage		0.628	
Overall solution consistency		0.948	

●Existence of center conditions; •Existence of contributing conditions.

## Conclusion and implications

Human capital, psychological capital, microsocial capital, team social capital, and macrosocial capital were integrated using combination thinking and QCA. The multiple concurrence factors and complicated influencing mechanism of the career success of scientific and technological innovation in universities were discussed. Some major conclusions were drawn, and are listed as follows:

1. There are four paths to generate the high career success of scientific and technological innovation talents in universities:

(1) Human capital*psychological capital*microsocial capital*macrosocial capital: the combination of high human capital, high psychological capital, high microsocial capital, and high macrosocial capital; (2) human capital*psychological capital*team social capital*macrosocial capital: the combination of high human capital, high psychological capital, high team social capital and high macrosocial capital; (3) human capital*microsocial capital*team social capital*macrosocial capital: the combination of high human capital, high microsocial capital, high team social capital, and high macrosocial capital; (4) psychological capital*microsocial capital*team social capital*macrosocial capital: the combination of high psychological capital, high microsocial capital, high team social capital and high macrosocial capital.

This shows that under the framework of career success driven by the three capital theories, there is no single best path for the career success of scientific and technological innovation talents in universities. Paths that trigger the same outcome are conditional and multidimensional. In the management practice of universities, managers need to first identify some of the three capital conditions that they already have, then compare the four stimulation paths, and finally supplement another part of the capital conditions, and seek the least resources to stimulate one of the paths, so as to improve the career success efficiency of the scientific and technological innovation talents in universities.

2. There are two paths to generate the non-high career success of scientific and technological innovation talents in universities:

(1)∼human capital*∼psychological capital*∼microsocial capital: the combination of low human capital, low psychological capital and low microsocial capital; (2)∼psychological capital*∼microsocial capital*∼team social capital*∼macrosocial capital: the combination of low psychological capital, low microsocial capital, low team social capital, and low macrosocial capital.

This means that psychological capital and micro-social capital are very important for the career success of scientific and technological innovation talents in universities. When psychological capital and micro-social capital are low, no matter how other conditions match, the career success of scientific and technological innovation talents in universities cannot be stimulated. Therefore, when managing scientific and technological innovation talents in universities, they should pay attention to the cultivation of their psychological capital to ensure that they have a positive psychological state and the confidence to overcome difficulties, so that they can maintain a high professional enthusiasm and commitment. On the other hand, because the research results show that, compared with macro social capital and team social capital, micro social capital plays a more important role in the career success of scientific and technological innovation talents in universities. Therefore, in the management practice, in addition to providing team platform support for scientific and technological innovation talents, encouraging and creating opportunities to help them establish a wider personal-level social resource network plays a more important role in helping their career success.

3. H4 shows the highest coverage, revealing that most scientific and technological innovation talents achieve career success by increasing psychological capital and using current surrounding social capital. Most samples conforming to this path are young people who have worked for a few years and have low titles. This indicates that scientific and technological innovation talents in the initial career stage tend to achieve career success through H4. In other words, talents join a good team and achieve career success by increasing their psychological capital, improving their social trust and using surrounding microscopic relation networks. At the same time, the marginal benefit from increasing human capital is relatively low.

4. By comparing the paths, high macrosocial capital is recognized as the center condition in all four paths that generate high employee career success. Going forward, the government must view increasing overall social trust as one of its primary job objectives in improving the career success of scientific and technological innovation talents.

## Limitations and future research directions

This study has some disadvantages. First, only the influences of four important variables of three aspects (human capital, psychological capital, and social capital) on the career success of scientific and technological innovation talents were discussed. However, other influencing factors and variables can be added in future studies to increase the universality of the research conclusions. Second, abundant qualitative data were supplemented to the discovered combinations. However, no deep qualitative analysis has been reported yet. In future, new theories of the career success of scientific and technological innovation talents can be studied in-depth by combining case studies. Finally, this study only collected static data due to data accessibility and could not collect cross-time data. With the increasing perfection and development of time series QCA, influences of capital environmental changes on the career success changes of scientific and technological innovation can be further discussed.

## Data availability statement

The original contributions presented in this study are included in the article/Supplementary material, further inquiries can be directed to the corresponding author.

## Author contributions

All authors listed have made a substantial, direct, and intellectual contribution to the work, and approved it for publication.
