# Supplementary material for: A configurational analysis on career success of scientific and technological innovation talents in universities
Source: Front Psychol. 2022 Dec 8;13:1068267. doi: 10.3389/fpsyg.2022.1068267 (PMC9773138; doi:10.3389/fpsyg.2022.1068267)
Supplement: Supplementary file 2 [file Data_Sheet_2.pdf]

Experts: Hello!

Thank you for taking the time out of your busy schedule to support us in doing this research. Scientific and technological innovation talents are the driving force for national development and social progress. This is a path combination retrospective analysis designed to study the career success of science and technology innovators in universities. Through this study, it is hoped to find more path configurations for the career success of sci-tech innovators, and to provide the basis for the personal career success and the policy support they need.

This questionnaire is left anonymous, there is no right or wrong answer, your answer is only for the overall statistical analysis, will not be open to others, please answer according to your true situation and feelings.

Part 1 Basic Information

Sex：1.male 2.female Marital Status：1. married 2. unmarried

Research Area：____

Age：_________ Seniority：___________years

Highest Degree Graduated College：__________________________

Highest education：_____________________________ Position：________________

Unit type:1.State-owned Enterprises 2.Private Enterprise 3.University and Research Institute 4.Sino-foreign Joint Venture Enterprise 5.Wholly Foreign-owned Enterprise(WFOE) 6. Institution 7.Other

Part 2 Human Capital Questionnaire

Please evaluate and judge your feelings with the following 22 descriptions according to your actual feelings and experience, and write down each corresponding number after each topic.The criteria for evaluation and judgment are as follows:

1.Very disagree 2.Disagree 3.Not sure 4.Agree 5.Very agree

1. You have the innovation ability.____

2. You have the ability to create value for your research projects.____

3. You have the ability to help your research team minimize the cost of project delivery.____

4. You have the ability to provide quality teaching or research services to your college or research team.____

5. You have the ability to develop new markets, new products, new services.____

6. You have the ability to develop the best industry-recognized scientific research products or services.____

7. You have the ability to improve the efficiency or productivity of your college or team.____

8. You have the ability to adapt your college or team to Party A's constantly changing demand for scientific research project capabilities.____

9. You have the ability to provide your team with competitive low-cost scientific research products and services.____

10. You have the ability to directly affect the satisfaction of the scientific research project service object (Party A).____

11. You have the ability to provide high-quality scientific research products or services.____

12. You have the ability to improve the organizational process.____

13. You are more competitive than any other labor force in the market.____

14. You're hard to replace by someone else.____

15. You have labor skills that your competitors don't have.____

16. Your labor skills within the industry are widely considered the best.____

17. Your job skills benefit from your working years.____

18. You have the ability that competitors are hard to buy.____

19. Your job skills are unique to your organization.____

20. Your competitors are hard to imitate or copy your job skills.____

21. Your job skills meet the requirements of your organization.____

22. It is your unique job skills that differentiate your organization from your competitors.____

The criteria for judging the following three questions are as follows:

1.Very disagree 2.Disagree 3.A little disagree 4.A little agree 5.Agree 6.Very agree

Please make a corresponding judgment based on your feelings, and write down the corresponding numbers after each question.

23. I have more professional and theoretical knowledge than my colleagues.____

24. I have more work experience than my colleagues.____

25. I have higher professional skills compared to my colleagues. ____

Part 3 Psychological Capital Questionnaire

Here are some sentences that describe how you may see yourself at present.Use the following 24 descriptions to evaluate and judge your feelings, and write down each corresponding number after each topic.The judgment criteria are as follows:

1.Very disagree 2.Disagree 3.A little disagree 4.A little agree 5.Agree 6.Very agree

1. I believe that I can analyze the long-term problems and find solutions.____

2. I was confident in stating things within the scope of my work in a meeting with the management.____

3. I believe I contributed to the company strategy.____

4. I believe I am able to help set goals within the scope of my work.____

5. I believe I am able to contact people outside the company (for example, suppliers, customers) and discuss issues.____

6. I believe I am able to present information to a group of colleagues.____

7. I can think of many ways to get out of the trouble that I find myself in at work.___

8. I am full of energy to complete my work goals at present.____

9. There are so many solutions to any problem.____

10. I think I am quite successful in my work now.____

11. I can come up with many ways to achieve my current work goals.____

12. I am achieving the work goals that I have set for myself currently.____

13. When I encounter setbacks at work, it was hard to recover from it and move on.__

14. I will solve the difficult problems at work in any case.____

15. If I have to do something at work, I can say that I can fight with it independently._

16. I am usually able to calmly handle the stress at work.____

17. I can survive the difficult times at work now， because I have gone through many hardships before.____

18. I feel that I can deal with many things at once in my current work.____

19. I usually expect the best results at work,when things are uncertain.____

20. If something must go wrong,it goes wrong even if I work wisely.____

21. I always see the bright side of things in my own work.____

22. I am optimistic about what happens in the future of my work.____

23. Things never have evolved as I wish it to be in my current work.____

24. I always believe that "behind the darkness is the light, do not be pessimistic" in my work.____

Part 4 Social Capital Questionnaire

（1）Microsocial capital

Please evaluate and judge your feelings with the following 23 descriptions according to your actual feelings and experience， and write down the corresponding number after each question.The criteria for evaluation and judgment are as follows:

1.Very disagree 2.Disagree 3.A little disagree 4.A little agree 5.Agree 6.Very agree

1．My circle of friends is very strong.____

2．I know a lot of people.____

3．I have a wide range of interpersonal contacts.____

4．I work with the people who have very different jobs.____

5．I work with the people who have very different nature of their work.____

6．I work with the people who have very different work area.____

7．I work with the people who have very different prestige.____

8．I meet people of high social status.____

9．I am close to some people who are in high positions.____

10．I have a lot of acquaintances who have great rights.____

11．I have a lot of acquaintances who are financially strong.____

12．My relatives are of great help to my job hunting.____

13．Relatives offer me valuable networks of relationships.____

14．I can get the guidance and help from my relatives at work.____

15．Relatives are helpful in my career development.____

16．My friends often give me advice at work.____

17．My friends often provide guidance and help in my work.____

18．My friend helped my career.____

19．People with an average relationship with me provide me with a lot of development opportunities.____

20．People with an average relationship with me provide me with a powerful network of relationships.____

21．People with an average relationship with me provide me some help when I am in trouble.____

22．People with an average relationship with me provide me with a lot of valuable information.____

23．People with an average relationship with me will help me develop my career.____

（2）Team social capital

Please evaluate and judge your feelings with the following 23 descriptions according to your actual feelings and experience， and write down the corresponding number after each question.The criteria for evaluation and judgment are as follows:

1.Very disagree 2.Disagree 3.A little disagree 4.A little agree 5.Agree 6.Very agree

1．Team (organization) members can do what they say.____

2．Team (organization) members believe that each other's commitment is reliable.___

3．Team (organization) members can help each other when their work is in trouble.__

4．Team (organization) members trust each other's working skills.____

5. Team (organization) members understand the terms that the other said.____

6. Everyone else can quickly understand the project issues described by members of the team (organization).____

7. Team (organization) members are very clear about the symbols, terms, and meanings of the professional areas involved in the project.____

8. Team (organization) members are familiar with the tools (software, processes, processes, etc.) involved in the project.____

9. Team (organization) holds dinner parties, fellowship and other informal activities.

10. Team (organization) holds some seminars.____

11. Team (organization) members communicate through phone calls, E-mail, chat software, etc.____

12. Team (organization) members talk in the company canteen, lounge, corridor and other informal occasions.____

13. Team (organization) members have a relatively consistent views on the development prospects of the project.____

14. Team (organization) members have a consistent understanding of the important things of the project (such as key technologies).____

15. Team (organization) members have more consistent views on the overall objectives of the team.____

16. Team (organization) members can reach consensus on decisions particularly critical to the project.____

17. The team (organization) director is always considerate and supportive in the future.____

18. The team (organization) director can treat the members very fairly.____

19. Team (organization) members believe that the director is competent for his / her position.____

20. Team (organization) members believe that what the director says is honest and credible.____

The following 21-23 question is "right or wrong", please judge the following description as "yes" or "non" according to your feelings.

21．Team (organization) members have a good personal relationship.____

22．Team (organization) members often discuss issues together.____

23．Team (organization) members have a lot of communication and cooperation in the work.____

（3）Macrosocial capital

Please evaluate and judge your feelings with the following 23 descriptions according to your actual feelings and experience， and write down the corresponding number after each question.The criteria for evaluation and judgment are as follows:

1.Very disagree 2.Disagree 3.Not sure 4.Agree 5.Very agree

1.In general, if you agree that the vast majority of people in this society can be trusted or not.

In addition, in your career, have you feel that any national, government, schools and other policies have helped to your career success?(Yes or No)_______

If so, please list them briefly:____

Part 5 Career Success Questionnaire

Please evaluate and judge your feelings with the following 11 descriptions according to your actual feelings and experience， and write down the corresponding number after each question.The criteria for evaluation and judgment are as follows:

1.Very disagree 2.Disagree 3.A little disagree 4.A little agree 5.Agree 6.Very agree

1．The unit sees me as a valuable resource.____

2．The unit thinks that I can create value for the organization because of my skills and experience.____

3．I have many development opportunities in the unit.____

4．I can easily have similar jobs in other units.____

5．With my skills and experience, I have plenty of jobs to choose from.____

6．With my skills and experience, other organizations will see me as a valuable resource.____

7. I am satisfied with the success of my career.____

8. I am satisfied with the progress I have made to meet my overall career goals.____

9. I am satisfied with the progress I have made to meet my income goals.____

10. I am satisfied with the progress I have made to meet my promotion goals.____

11. I am satisfied with the progress I have made to meet the goal of acquiring new skills.____
